# The Interaction between Auxin and Nitric Oxide Regulates Root Growth in Response to Iron Deficiency in Rice

**DOI:** 10.3389/fpls.2017.02169

**Published:** 2017-12-22

**Authors:** Huwei Sun, Fan Feng, Juan Liu, Quanzhi Zhao

**Affiliations:** College of Agronomy, Collaborative Innovation Center of Henan Grain Crops, Laboratory of Rice Biology in Henan Province, Henan Agricultural University, Zhengzhou, China

**Keywords:** auxin, iron deficiency, nitric oxide, rice, root

## Abstract

Fe deficiency (-Fe) is a common abiotic stress that affects the root development of plants. Auxin and nitric oxide (NO) are key regulator of root growth under -Fe. However, the interactions between auxin and NO regulate root growth in response to Fe deficiency are complex and unclear. In this study, the indole-3-acetic acid (IAA) and NO levels in roots, and the responses of root growth in rice to different levels of Fe supply were investigated using wild type (WT), *ospin1b* and *osnia2* mutants. -Fe promoted LR formation but inhibited seminal root elongation. IAA levels, [^3^H] IAA transport, and expression levels of *PIN1a-c* genes in roots were reduced under -Fe, suggesting that polar auxin transport from shoots to roots was decreased. Application of IAA to -Fe seedlings restored seminal root length, but not LR density, to levels similar to those under normal Fe (+Fe), and the seminal root length was shorter in two *ospin1b* mutants relative to WT under +Fe, but not under -Fe, confirming that auxin transport participates in -Fe-inhibited seminal root elongation. Moreover, -Fe-induced LR density and -Fe-inhibited seminal root elongation paralleled NO production in roots. Interestingly, similar NO accumulation and responses of LR density and root elongation were observed in *osnia2* mutants compared to WT, and the higher expression of *NOA* gene under -Fe, suggesting that -Fe-induced NO was generated via the NO synthase-like pathway rather than the nitrate reductase pathway. However, IAA could restore the functions of NO in inhibiting seminal root elongation, but did not replace the role of NO-induced LR formation under -Fe. Overall, our findings suggested that NO functions downstream of auxin in regulating LR formation; NO-inhibited seminal root elongation by decreasing meristem activity in root tips under -Fe, with the involvement of auxin.

## Introduction

Iron (Fe) is a major nutrient needed for plant growth and development, and Fe deficiency dramatically affects plant growth, formation, and productivity (Guerinot and Yi, 1994). Plants absorb Fe from soil. Despite the high level of Fe in soil, the solubility and availability of Fe are low in calcareous soil, which accounts for 30% of cultivated land worldwide. Fe deficiency reduces both the growth and productivity of plants (Guerinot and Salt, 2001); therefore, an understanding of the responses of plants to Fe deficiency is important to improve growth of crops under adverse conditions.

To adapt to Fe-deficient (-Fe) conditions, plants have developed multiple strategies to absorb Fe, such as the improvement of Fe uptake by regulating the expression of Fe-related genes (Yang et al., 2013) or a change in root morphology (Wang et al., 2010; Jin et al., 2011; Chen and Kao, 2012; Meng et al., 2012; Zhai et al., 2016). Jin et al. (2009) suggested that Fe deficiency increased the development of root hairs in tomato. A similar finding was reported in *Malus xiaojinensis* by Zhai et al. (2016). Fe deficiency induces root branching in several plants (Moog et al., 1995; Dasgan et al., 2002; Jin et al., 2008, 2011; Long et al., 2010). Moreover, under Fe deficiency, the formation of cluster roots was induced in white lupin (Wang et al., 2010; Meng et al., 2012) and the primary root length was inhibited in tomato (Jin et al., 2011). Although the responses of roots to Fe stress at the physiological level are well documented, the mechanisms underlying these changes are unclear.

Root growth and development of plants is regulated by environmental conditions and intrinsic factors (e.g., phytohormones). Auxin plays a critical role in root growth of plants (Friml, 2003; Sun et al., 2014). Most auxin is synthesized in aboveground tissues, such as shoot apices and young leaves, by *YUCCA* family genes (Ljung et al., 2001; Stepanova et al., 2011; Zhao, 2012) and then redistributed by auxin-influx carriers, such as AUX1/LAX family proteins, and auxin-efflux carriers, including ABCB/PGP and PIN family proteins (Friml, 2003; Blakeslee et al., 2005; Zazimalova et al., 2010; Peret et al., 2012). PIN proteins are the major auxin efflux carriers in plants (Friml, 2003; Wisniewska et al., 2006). Auxin levels are regulated under varying Fe supplies (Jin et al., 2007; Chen et al., 2010; Jin et al., 2011). Studies on red clover (*Trifolium pratense*), *Arabidopsis*, and tomato also demonstrated the accumulation of auxin in Fe-deficient roots (Jin et al., 2007, 2011; Chen et al., 2010). Several lines of evidence suggest that auxin is involved in plant root growth and the expression of Fe-uptake genes under Fe deficiency condition (Jin et al., 2011; Giehl et al., 2012; Blum et al., 2014; Rai et al., 2015). However, the mechanism of auxin in regulating root growth under Fe deficiency is not available.

Nitric oxide (NO), a signaling molecule, is involved in a variety of physiological processes in response to Fe deficiency. For example, NO production contributes to the improvement of Fe acquisition and homeostasis by regulating the expression of Fe-related genes under Fe-deficient condition (Graziano and Lamattina, 2007; Chen et al., 2010; García et al., 2010; Meiser et al., 2011; Yang et al., 2013). Furthermore, NO also involvement in root development is regulated under Fe deficiency in several plant species (Chen et al., 2010; Jin et al., 2011; Meng et al., 2012; Zhai et al., 2016). A higher NO level in Fe-deficient roots enhanced root branching in tomato (Jin et al., 2011). Similarly, the endogenous NO concentration in Fe-deficient roots positively regulated the formation of cluster roots in white lupin (Meng et al., 2012) and enhanced root hair growth in *M. xiaojinensis* (Zhai et al., 2016). However, the mechanism of NO in regulation of root growth under Fe deficiency requires further investigation.

The roles of auxin and NO in regulation of root growth are closely linked (Correa-Aragunde et al., 2004; Fernández-Marcos et al., 2011; Jin et al., 2011; Chen and Kao, 2012). Chen and Kao (2012) reported that the NO donor Sodium nitroprusside (SNP) and exogenous application of the auxin indole-3-butyric acid (IBA) increased the NO level in roots, and that the effects of SNP and IBA on lateral root (LR) formation were significantly inhibited by the NO scavenger 2-(4-carboxyphenyl)-4,4,5,5-tetramethylimidazoline-1-oxyl-3-oxide (cPTIO), suggesting that NO acted downstream of auxin in regulating LR formation in rice. However, the roles of NO and auxin in regulating root growth differed between elongation of roots and LR formation. For example, NO inhibited the elongation of primary roots by reducing acropetal auxin transport in *Arabidopsis* (Fernández-Marcos et al., 2011), suggesting that NO regulates root elongation based on the auxin levels in root tips (RT). Therefore, the interaction between auxin and NO in regulating root growth is complex and unclear. Jin et al. (2011) demonstrated that NO acted downstream of auxin in regulating root branching under Fe deficiency. Further research is needed to explore the links between NO and auxin in the control of root growth in response to Fe deficiency. Moreover, most studies on regulation of root growth by Fe deficiency involved dicotyledons, for instance, *Arabidopsis* and tomato; few have focused on monocotyledons.

Rice, one of the important food crops in the word, is an ideal model for studying root growth and development in response to nutrient deficiency because it has a range of well-characterized related mutants (Chen and Kao, 2012; Chen et al., 2013; Sun et al., 2014, 2015, 2016). In this study, we evaluated the LR density and seminal root length of rice seedlings and measured auxin concentrations, [^3^H] indole-3-acetic acid (IAA) transport, and NO levels under +Fe and -Fe. Rice plants were transformed with the auxin reporter construct *DR5::GUS* and used to identify the pattern of auxin distribution in response to Fe deficiency. The expression levels of auxin- and NO-related genes were determined by real-time quantitative reverse transcription (qRT)-PCR. In addition, we examined the effects of exogenous application of the auxin IAA, the auxin polar transport inhibitor *N*-1-naphthylphthalamic acid (NPA), SNP, and cPTIO on LR formation and seminal root elongation of rice seedlings. Finally, the mechanisms by which NO and auxin regulate root growth in response to Fe deficiency in rice were explored.

## Materials and Methods

### Plant Materials

The Nipponbare ecotype of rice (Oryza sativa L.) was used in this study. And two T-DNA insertion *osnia2* mutant lines (*osnia2-1* and *osnia2-2*) and two *ospin1b* mutant lines (*ospin1b-1* and *ospin1b-2*) in the Dongjin ecotype (Japonica rice) were obtained from the Rice Functional Genomics Express Database (RiceGE; Pohang City, Korea). Two *osnia2* mutant lines were identified by Sun et al. (2016) and two *ospin1b* mutant lines were identified in Supplementary Figures 4A,B.

### Plant Growth

Rice seedlings were grown in a greenhouse at day/night temperatures of 30°C/18°C under natural light. One-week-old seedlings of uniform size and vigor were transplanted into holes in a lid placed atop pots. Nutrient solutions ranging from one-quarter, one-third, one-half to full strength were applied for 1 week, followed by full-strength nutrient solution for 1 week under any nutrient treatments. Pots containing Fe treatments were filled with solutions lacking Fe or receiving varying Fe concentrations (0, 2.5, 5, 10, and 20 μM). Normal nutrition (+Fe) contained 20 μM Fe-EDTA, and Fe-deficient nutrition contained 0 μM Fe-EDTA. The chemical composition of International Rice Research Institute (IRRI) nutrient solution was (mM): 2.5 NH_4_NO_3_, 0.3 KH_2_PO_4_, 0.35 K_2_SO_4_, 1.0 CaCl_2_, 1.0 MgSO_4_⋅7H_2_O, 0.5 Na_2_SiO_3_, and (μM) 9.0 MnCl_2_, 0.39 (NH_4_)_6_Mo_7_O_24_, 20.0 H_3_BO_3_, 0.77 ZnSO_4_, and 0.32 CuSO_4_; pH 5.5.

The treatments of 0–1000 nM IAA (Indole-3-acetic acid, dissolved in 1 M NaOH), 0–1000 nM NPA (*N*-1-naphthylphalamic acid, dissolved in dimethyl sulfoxide, DMSO), 0–30 μM SNP (Sodium nitroprusside, dissolved in water), 25 μM Tu (Tungstate, dissolved in water), 25 μM tungstate (Tu, dissolved in water), 100 μM cPTIO (2-(4-carboxyphenyl)-4,4,5,5-tetramethylimidazoline-1-oxyl-3-oxide, dissolved in water), and 100 μM L-NAME (NG-nitro-L-arginine methyl ester dissolved in water) were applied by the plant-growth media. Seven-day-old rice seedlings were grown in nutrient solutions containing +Fe and -Fe follow the Plant growth conditions above.

### Measurement of Root System Architecture

Seminal roots were significantly longer than adventitious roots in this study. The responses of seminal roots to different treatments were similar to those of adventitious roots in our preliminary experiment. Therefore, seminal roots and LRs of seminal roots were used to study the effects of Fe at two concentrations on the rice root system. Seminal root length was measured using a ruler. LR density was calculated as the LR number divided by the seminal root length.

To visualize the formation of LR primordia, *pDR5::GUS*, a specific reporter that contains seven repeats of a synthetic auxin response element and can reflect the *in vivo* auxin levels (Ulmasov et al., 1997), were transformed to rice plants. After staining of roots in β-glucuronidase (GUS) buffer for 2 h, the number of LR primordia was counted using a stereomicroscope (Olympus SZX16) according to Sun et al. (2015).

### Determination of Iron Concentration

Extraction of water-soluble iron was analyzed as described (Cassin et al., 2009). The 0.5 g plant tissues (shoots and roots) of 14-day-old seedlings was grinded with liquid N_2_, and extracted by 5 volumes of distilled water. The samples were centrifuged at 2000 r/min speed. The supernatant was recovered and the pellet was resuspended with the equivalent volume of distilled water. Fe concentration was measured by inductively coupled plasma mass spectrometry.

### Determination of IAA

Indole-3-acetic acid concentrations in the first leaves, shoot-root junctions, and roots were analyzed as described previously (Lu et al., 2009). Fresh samples were weighed to 0.5g and then immediately frozen in liquid N_2_. Free IAA levels were determined by high-performance liquid chromatography (HPLC) according to Lu et al. (2009).

To assess the auxin distribution in rice plants, rice plants were transformed with the *pDR5::GUS* construct by *Agrobacterium tumefaciens* (strain EHA105). The *pDR5::GUS* construct was kindly provided by Professor Ping Wu of Zhejiang University, Hangzhou, China. The first leaves, shoot–root junctions, and roots were used for histochemical GUS staining. The first leaves were soaked with ethanol prior to observation to eliminate chlorophyll pigmentation. Plants were stained for *GUS* activity in the first leaf for 24 h and in the junction of root and shoot, and roots for 2 h at 37°C. Stained plant tissues were photographed using a stereomicroscope (Olympus SZX16) equipped with a color CCD camera.

### [^3^H] IAA-Transport

Shoot-to-root auxin transport in rice plants was assayed according to Song et al. (2013). Polar transport of [^3^H] IAA was assayed in root samples under +Fe (20 μM) and -Fe (0 μM). [^3^H] IAA solution contained 0.5 μM [^3^H] IAA (20 Ci mmol^-1^) in 2% dimethylsulphoxide (DMSO), 25 mM MES (pH 5.2), and 0.25% agar.

Shoot-to-root auxin transport in rice plants was measured as follows. [^3^H] IAA solution (20 μL) was applied to the cut surface as soon as removing rice shoots 2 cm above the shoot bases. The two root segments, namely the RT and all the LR region of seminal root, were weighed and incubated in 4 mL of scintillation solution for 18 h in darkness. [^3^H] IAA radioactivity was detected using a multipurpose scintillation counter (LS6500; Beckman–Coulter, Fullerton, CA) (Song et al., 2013).

### Cortical Cell Length Analysis

The method of cortical cell length analysis as described previously (Jia et al., 2008). The cortical cell visualized with a microscope (Olympus SZX16) equipped with a color CCD camera. The average cortical cell length of the maturation zone of seminal roots was obtained using a mixture of cortical cells with eight replicates in the maturation zone (on average per longitudinal section).

### *pCYCB1;1::GUS* construct

The *pCYCB1;1::GUS* fusion construct was constructed as described by Colón-Carmona et al. (1999) and was kindly provided by Professor Chuanzao Mao of Zhejiang University, Hangzhou, China. The *pCYCB1;1::GUS* fusion construct was transformed into rice plants. Plants were stained for *GUS* activity in the RTs for 2 h at 37°C. The RTs were subjected to histochemical *GUS* staining and photographed using a microscope (Olympus SZX16) equipped with a color CCD camera.

### Measurement of NO Levels in Roots

Nitric oxide was imaged using 4-amino-5-methylamino-2′7′-difluorofluorescein diacetate (DAF-FM DA) and an epifluorescence microscope. The roots were soaked with 10 μM DAF-FM DA in 20 mM HEPES-NaOH buffer (pH 7.5). After being kept in the dark for 30 min, the roots were washed three times in fresh buffer and immediately observed using a stereomicroscope (Olympus SZX16, excitation 488 nm, emission 495–575 nm) equipped with a color CCD camera. The green fluorescence intensity was quantified as described previously (Guo and Crawford, 2005) with Photoshop software (Adobe Systems, San Jose, CA, United States).

### Measurement of Nitrate Reductase (NR) Activity in Roots

Nitrate reductase (NR) activity in rice roots was measured according to Ogawa et al. (1999). The assay mixture contained 25 mM K_3_PO_4_ buffer (pH 7.5), 10 mM KNO_3_, 0.2 mM NADH, 5 mM NaHCO_3_, and 5 μL of extract from a final volume of 0.5 mL. The assays were conducted at 30°C for 15 min. The reaction was terminated by adding 50 μL of 0.5 M Zn(CH_3_COO)_2_ and excess NADH was oxidized by adding 50 μL of 0.15 mM phenazine methosulphate. The mixture was centrifuged at 10,000 *g* for 5 min. The NO_2_^-^ level was quantified by combining 500 μL of supernatant with 250 μL of 1% sulphanylamide prepared in 1.5 N HCl and 250 μL of 0.02% *N*-(1-naphthyl)ethylene-diamine dihydrochloride, and the absorbance at 540 nm was read using a spectrophotometer.

### qRT-PCR

Total RNA was isolated from the first leaves and roots of wild type (WT) (*Nipponbare*) under +Fe and -Fe supplies, WT (Dongjin) and two *ospin1b* mutant lines under +Fe condition for 14 days. The methods of RNA extraction, reverse transcription, and qRT-PCR were as given previously Jia et al. (2011). The primer sets for *YUCCA1-8, PIN1-10, NOA, NIA1, NIA2, CYCB1;1, YSL15*, and *IRT1* are listed in Supplementary Tables 1–4, which are available online.

### Data Analysis

Data were pooled for calculation of means and standard errors (SE) and subjected to one-way analysis of variance (ANOVA), followed by Ryan–Eynot–Gabriel–Welch *F* test at *P* < 0.05 to determine the statistical significance of differences between treatments. All statistical evaluations were conducted using SPSS (version 11.0) statistical software (SPSS Inc., Chicago, IL, United States).

## Results

### Fe Deficiency Inhibited Seminal Root Elongation, But Increased LR Density

**FIGURE 1:**
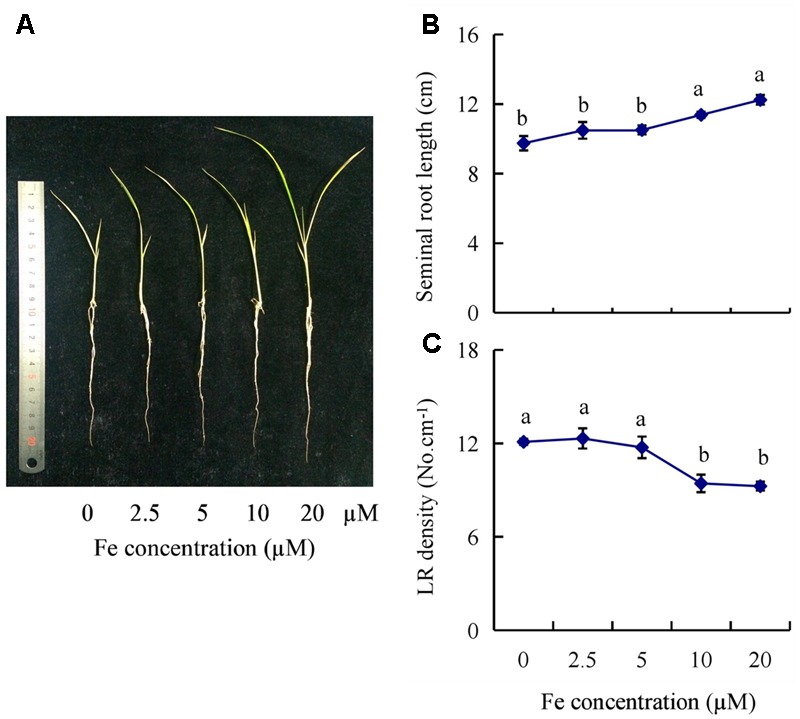
Effects of iron (Fe) availability on root morphology in rice seedlings. Seedlings were grown in hydroponic media containing varying concentrations of Fe for 14 days. **(A)** The morphology of the rice plants. **(B)** The length of seminal root. **(C)** Lateral root (LR) density. Data are means ± SE and bars with different letters indicate significant difference at *P* < 0.05 tested with ANOVA.

The seminal root length of rice plants decreased significantly as Fe concentration decreased from 20 to 0 μM (**Figures 1A,B**). The seminal root length was decreased by 15% in 2.5 μM Fe and by approximately 21% in 0 μM Fe (-Fe) compared to that under the sufficient Fe condition (+Fe, 20 μM) (**Figure 1B**). The LR density was increased by 30% in -Fe relative to +Fe (**Figure 1C**). Compared with +Fe, the Fe concentrations of shoot and root were decreased by 53% and 61% under -Fe, respectively (Supplementary Figure 1A). And the relative levels of *YSL15* and *IRT1* gene expression were significantly enhanced by -Fe compared with +Fe (Supplementary Figure 1B).

### Fe Deficiency Increased Auxin Levels in the First Leaves, But Decreased Them in Roots

**FIGURE 2:**
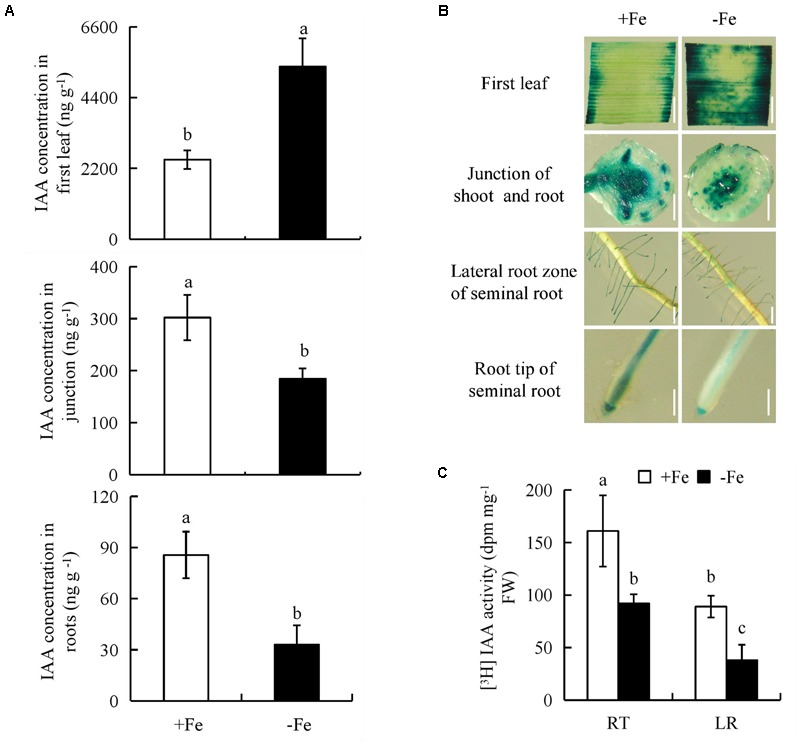
Indole-3-acetic acid (IAA) concentration and histochemical localization of *DR5::GUS* activity and [^3^H]IAA transport in rice seedlings. Rice seedlings were grown in hydroponic media containing -Fe (0 μM) and +Fe (20 μM) for 14 days. **(A)** IAA concentration in first leaf, the junction of root and shoot, and roots. **(B)** *DR5::GUS*, a specific reporter that contains seven repeats of a highly active synthetic auxin response element and can reflect the *in vivo* auxin level. Plants were stained for *GUS* activity in the first leaf for 24 h and in other regions for 2 h at 37°C. Bar = 1 mm. **(C)** Root tip [The root tip (RT) of seminal root]; LR zone. Data are means ± SE and bars with different letters indicate significant difference at *P* < 0.05 tested with ANOVA.

To determine whether the auxin levels is regulated in rice plants under -Fe, we measured endogenous IAA concentrations in the first leaves, shoot-root junctions, and roots of rice seedlings; these were increased by 117% and decreased by 39% and 61%, respectively, under -Fe relative to +Fe (**Figure 2A**).

We investigated the effects of -Fe and +Fe on auxin status in rice using transgenic plants expressing the *pDR5::GUS* construct. *DR5::GUS* expression was more widely distributed in the first leaves, but less widely in the shoot-root junctions and roots under -Fe relative to +Fe (**Figure 2B**). This was consistent with the findings on IAA concentrations. Compared with +Fe, [^3^H] IAA transport from shoots to roots was significantly decreased by -Fe, which led to a lower [^3^H] IAA activity in the LR region and RTs (**Figure 2C**). Thus, it could be inferred that the polar auxin transport was decreased from shoots to roots under -Fe.

### Auxin Is Involved in Seminal Root Elongation But Not LR Formation

**FIGURE 3:**
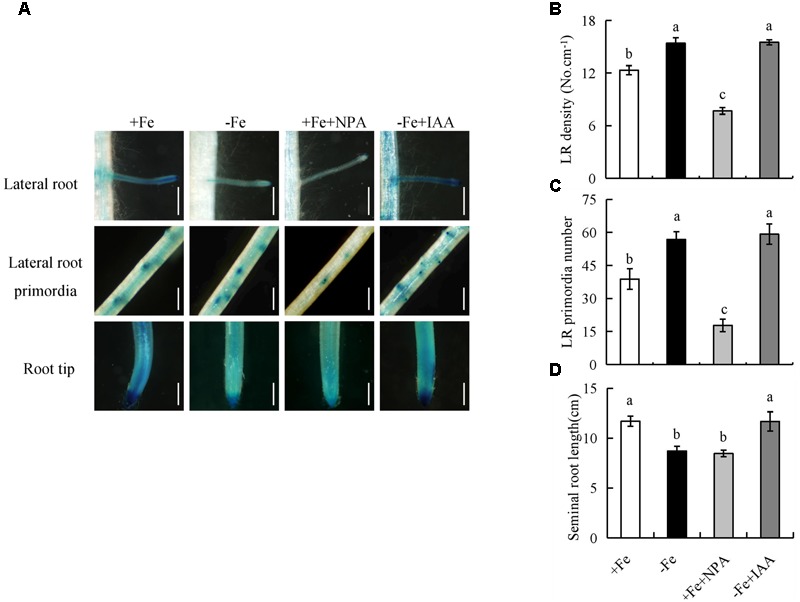
Histochemical localization of *DR5::GUS* activity and root morphology of seminal root in rice seedlings. Rice seedlings were grown in hydroponic media containing -Fe (0 μM) and +Fe (20 μM) in addition to IAA (100 nM) and NPA (100 nM) for 14 days. **(A)** Plants were stained for *GUS* activity for 2 h at 37°C. Bar = 1 mm. **(B)** LR density. **(C)** LR primordial number. **(D)** Seminal root length. Data are means ± SE and bars with different letters indicate significant difference at *P* < 0.05 tested with ANOVA.

We examined LR density, LR primordia number, and seminal root length after application of NPA and IAA to determine whether the decreased auxin level resulting from -Fe was responsible for morphological changes of underground roots. The LR density and seminal root length of rice plants supplied with 0–1000 nM NPA under +Fe and 0–1000 nM IAA under -Fe were measured (Supplementary Figure 2). Treatment of rice plants with NPA (100 nM) under +Fe markedly decreased *DR5::GUS* expression in roots (LR region and RT) and seminal root length to levels similar to those under -Fe, but did not mimic the changes in LR density and LR primordia under -Fe (**Figures 3A–C**). Moreover, application of IAA (100 nM) under -Fe also increased *DR5::GUS* expression in roots (LR region and RT) and seminal root length to levels similar to those under +Fe (**Figure 3D**), but LR density and LR primordia were unaffected (**Figures 3A–C**), suggesting that seminal root elongation rather than LR formation is regulated by auxin levels under -Fe.

### The Expression of *OsPIN1b* in WT and the Root Morphology of *ospin1b* Mutants Are Regulated under Fe Deficiency

**FIGURE 4:**
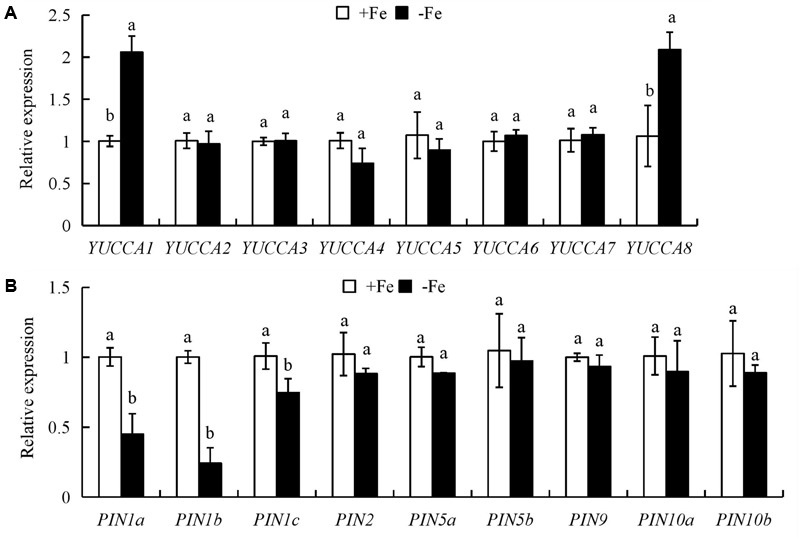
Quantitative reverse transcription (qrt)-PCR analysis of *YUCCA* and *PIN* family genes in rice seedlings. Rice seedlings were grown in hydroponic media containing -Fe (0 μM) and +Fe (20 μM) for 14 days. **(A)** The expression of *OsYUCCA* genes in the first leaf. **(B)** The expression of *OsPIN* genes in roots. Relative mRNA levels were normalized for individual gene relative to Os*ACT*. Data are means ± SE and bars with different letters in the same gene indicate significant difference at *P* < 0.05 tested with ANOVA.

We analyzed the expression of auxin biosynthesis genes (*YUCCA1-8*) in the first leaves and auxin transport genes (*PIN1-10*) in roots. The expression levels of *YUCCA1* and *YUCCA8* in the first leaves were significantly upregulated by -Fe relative to +Fe (**Figure 4A**); however, compared with +Fe, the expression levels of *PIN1a*-*c* in roots were downregulated under -Fe (**Figure 4B**).

The expression of nine *OsPIN* family genes was analyzed in roots of rice plants (Supplementary Figure 3A). The expression level of *OsPIN1b* was the highest among the nine *OsPIN* genes in rice root, consistent with findings in other rice cultivars (Wang et al., 2009). Therefore, *OsPIN1b* was used as a target gene in subsequent analyses. To confirm the expression of *OsPIN1b* in response to -Fe, we determined the *GUS* expression level driven by the *OsPIN1b* promoter in roots (Supplementary Figure 3B). Compared with +Fe, the expression level of *pPIN1b::GUS* in roots was significantly reduced under -Fe (Supplementary Figure 3B).

The *ospin1b-1* and *ospin1b-2* mutants and WT (Dongjin) were used to assess the sensitivity of root growth to -Fe. Molecular characterisation of the two homozygous T-DNA insertion *ospin1b-1* and *ospin1b-2* mutants revealed that the T-DNAs were inserted into the promoter and 5′-URT regions, respectively (Supplementary Figures 4A,B). Compared with the WT plants, *OsPIN1b* expression was almost completely suppressed in both *ospin1b* lines (Supplementary Figure 4C).

**FIGURE 5:**
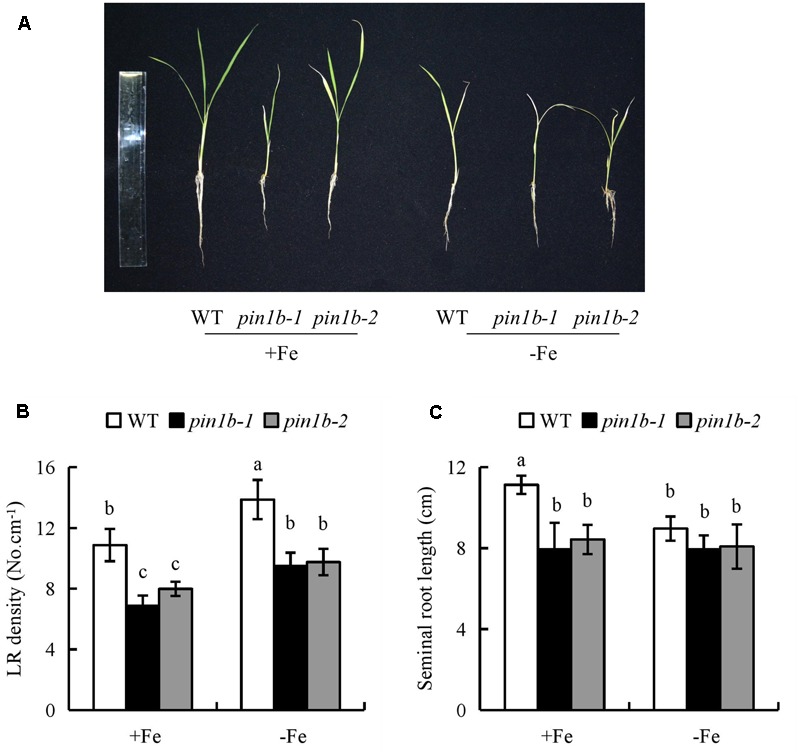
The root morphology in wild-type (WT, Donjin) and *ospin1b* mutants. Seedlings were grown in hydroponic media -Fe (0 μM) and +Fe (20 μM) for 14 days. **(A)** The morphology of the rice plants; **(B)** LR density. **(C)** The length of seminal root. Data are means ± SE and bars with different letters indicate significant difference at *P* < 0.05 tested with ANOVA.

The IAA concentration and [^3^H] IAA transport in roots of the *ospin1b-1* mutant did not differ between +Fe and -Fe (Supplementary Figure 5). Compared with WT plants, the LR density of two *ospin1b* mutants was reduced under +Fe and -Fe, and the seminal root length was shorter in the two *ospin1b* mutants under +Fe, but had a similar level to that in WT under -Fe. However, the density of two *ospin1b* mutants was increased under -Fe relative to +Fe (**Figure 5**). These findings confirmed that the seminal root elongation rather than LR formation is regulated by decreasing auxin polar transport under -Fe.

### NO Might Be Generated via NOS-Like and Is Involved in LR Formation and Seminal Root Elongation under -Fe

**FIGURE 6:**
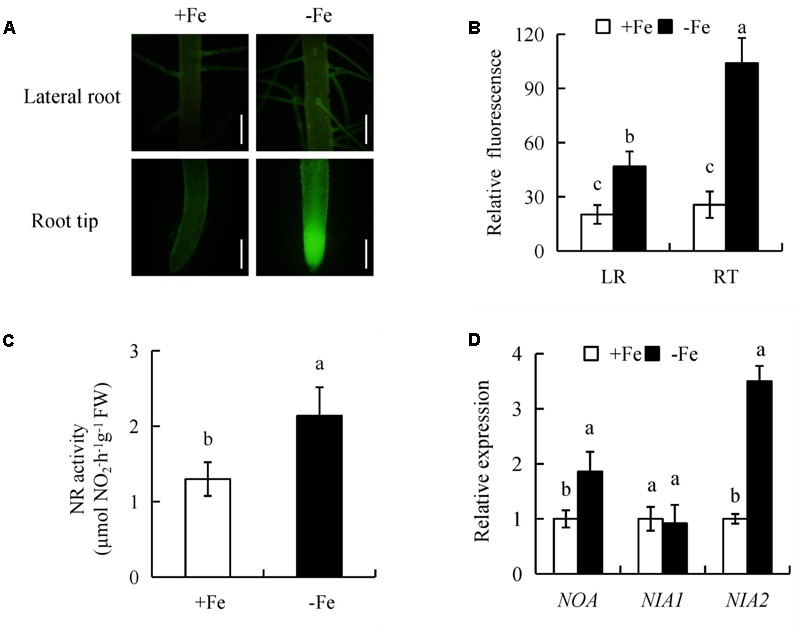
Accumulation of nitric oxide (NO), relative nitrate reductase (NR) activity, and qRT-PCR analysis of *NO-associated* (*NOA*), *NIA1, NIA2* genes in the rice seedlings. Seedlings were grown in hydroponic medium containing -Fe (0 μM) and +Fe (20 μM) for 14 days. **(A,B)** Photographs of NO production shown as green fluorescence in the seminal roots **(A)**, and NO production expressed as fluorescence intensity relative to the roots **(B)**. **(C)** Relative nitrate reductase (NR) activity in the roots. **(D)** Relative expression of *NOA, NIA1*, and *NIA2* in roots. Bar=1 mm. Data are means ± SE and bars with different letters indicate significant difference at *P* < 0.05 tested with ANOVA.

To determine whether NO participates in LR formation and seminal root elongation under -Fe, we evaluated NO-associated green fluorescence in seminal roots (LR region and RT) (**Figure 6**). In comparison with +Fe, NO-associated green fluorescence signals in RTs and LR region were increased under -Fe (**Figures 6A,B**), suggesting that NO production in roots is induced by -Fe.

NR activity in rice roots was assessed under +Fe and -Fe. Compared with +Fe, -Fe resulted in a significant increase in NR activity of 64% in roots (**Figure 6C**). qRT-PCR analysis showed that *NIA2* expression was significantly upregulated under -Fe relative to +Fe. However, the expression of *NIA1* had no differences between +Fe and -Fe, suggesting that -Fe increased NR activity via *NIA2*. Compared with +Fe, the transcription level of NO-associated (*NOA*) (a homolog of *NOA1* in *Arabidopsis*) in roots increased by 86% under -Fe (**Figure 6D**). Therefore, these results suggested that NO production maybe enhanced by NR and NOS-like pathways under -Fe.

**FIGURE 7:**
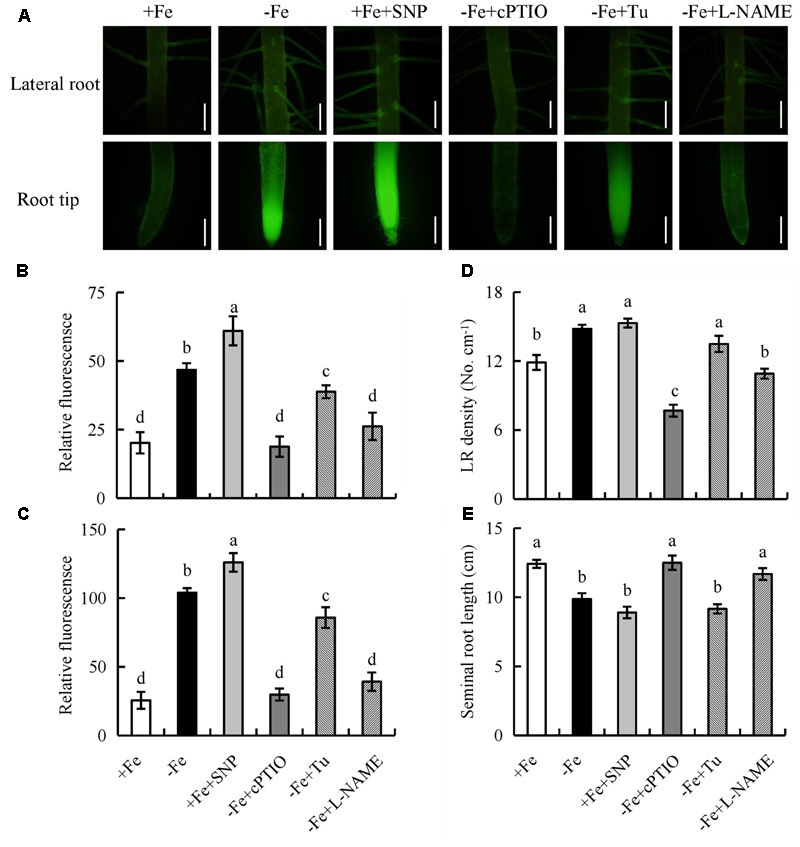
Accumulation of NO and root morphology in the rice seedlings. Seedlings were grown in hydroponic medium containing -Fe (0 μM) and +Fe (20 μM) in addition to SNP (30 μM), cPTIO (80 μM), Tungstate (Tu; 25 μM) and L-NAME (100 μM) for 14 days. **(A–C)** Photographs of NO production shown as green fluorescence in the seminal root **(A)**, and NO production expressed as fluorescence intensity relative to LR **(B)** and RT **(C)**. **(D)** LR density. **(E)** Seminal root length. Bar=1 mm. Data are means ± SE and bars with different letters indicate significant difference at *P* < 0.05 tested with ANOVA.

Under -Fe, application of the NR inhibitor Tu (25 μM) slightly decreased the NO-associated green fluorescence signal in roots, while no changes in seminal root length or LR density were observed. However, after treatment of rice plants with the NOS inhibitor L-NAME (100 μM) under -Fe, the NO-associated green fluorescence in roots, seminal root length, and LR density decreased markedly to levels similar to those under +Fe (**Figure 7**). Therefore, NO was generated from NOS-like rather than NR under -Fe.

Two *osnia2* mutant lines (*osnia2-1* and *osnia2-2*) were identified by Sun et al. (2016). Fe deficiency significantly increased NO accumulation in the roots. The LR density and seminal root length of both mutant lines to levels similar to those of WT plants under +Fe and -Fe (Supplementary Figures 6, 7). The results using *osnia2* mutants, which have lower *NR* activity, confirmed that NO production did not result from NIA2-dependent NR pathway under -Fe.

We next examined the effect on rice root morphology of the NO donor SNP and the NO scavenger cPTIO under -Fe. Application of 0–30 μM SNP significantly increased the NO-associated green fluorescence signal in roots under +Fe. Application of SNP at 30 μM increased the NO-associated green fluorescence signal and LR density, but decreased the seminal root length to levels similar to those under -Fe (Supplementary Figure 8). Application of cPTIO under -Fe markedly decreased the NO-associated green fluorescence signal and LR density, but increased the seminal root length to levels similar to those under +Fe (**Figure 7**). Thus, NO production in rice roots is enhanced by -Fe and is involved in LR formation and seminal root elongation.

### The Interaction of Auxin and NO Regulates Root Growth in Response to -Fe

**FIGURE 8:**
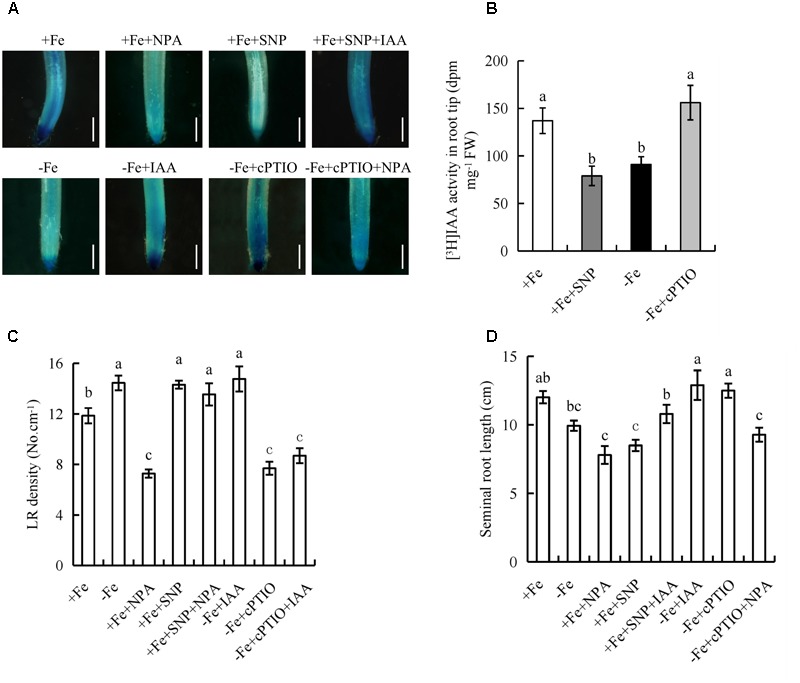
Histochemical localization of *DR5::GUS* activity, [^3^H]IAA transport and root morphology of seminal root in rice seedlings. Rice seedlings were grown in hydroponic media containing -Fe (0 μM) and +Fe (20 μM) in addition to IAA (100 nM), NPA (100 nM), SNP (30 μM), and cPTIO (80 μM) for 14 days. **(A)** Plants were stained for *GUS* activity in RT for 2 h at 37°C. Bar = 1 mm. **(B)** [^3^H] IAA transport. **(C)** LR density. **(D)** Seminal root length. Data are means ± SE and bars with different letters indicate significant difference at *P* < 0.05 tested with ANOVA.

Both NO and auxin are involved in the regulation of root growth in response to Fe deficiency, so we investigated the effect of their interaction. The inhibitory effect of NPA on LR formation was reversed by SNP under +Fe, but the inhibitory effect of cPTIO on LR formation was unaffected by IAA under -Fe (**Figures 8C,D**), suggesting that NO acts downstream of auxin in regulating LR formation under -Fe. We investigated the effects of NO on auxin status in rice using transgenic plants expressing the *DR5::GUS* construct and [^3^H]IAA transport. Application of SNP (30 μM) under +Fe decreased *DR5::GUS* expression and [^3^H]IAA activity in RTs to levels similar to those under -Fe, and application of cPTIO under -Fe markedly increased *DR5::GUS* expression and [^3^H]IAA activity in RTs to levels similar to those under +Fe (**Figures 8A,B**), which confirmed that NO decreases auxin levels in RTs under Fe deficiency. The application of both SNP and IAA increased the NO-associated green fluorescence signal in RTs to a level similar to that caused by SNP alone under -Fe. Moreover, the application of both cPTIO and NPA decreased the NO-associated green fluorescence signal in RTs to a level similar to that caused by cPTIO alone under +Fe (Supplementary Figure 9). Therefore, IAA did not affect induction by SNP of NO-associated green fluorescence in RTs. However, the inhibitory effect of SNP on auxin level in RTs and seminal root elongation was partly reversed by IAA under +Fe. cPTIO increased the auxin level in RTs, and seminal root length was restored by NPA under -Fe (**Figures 8C,D**), suggesting that the role of NO in regulating root elongation is dependent on the auxin levels in RTs of rice plants under Fe deficiency.

Application of IAA to the *ospin1b* mutant, increased the seminal root length to the levels similar to that in WT (Supplementary Figure 10), however, the seminal root length of *ospin1b* mutant gave no response to SNP and cPTIO under +Fe and -Fe, respectively. Application of +SNP+IAA increased the seminal root length to a level similar to that caused by +IAA alone under +Fe, and the seminal root length gave no response to treatment with +cPTIO+NPA under -Fe. These results further confirmed that the root elongation is dependent on the auxin levels in RTs of rice plants under Fe deficiency.

### Fe Deficiency Decreases Root Meristem Activity, But Not Cell Elongation

**FIGURE 9:**
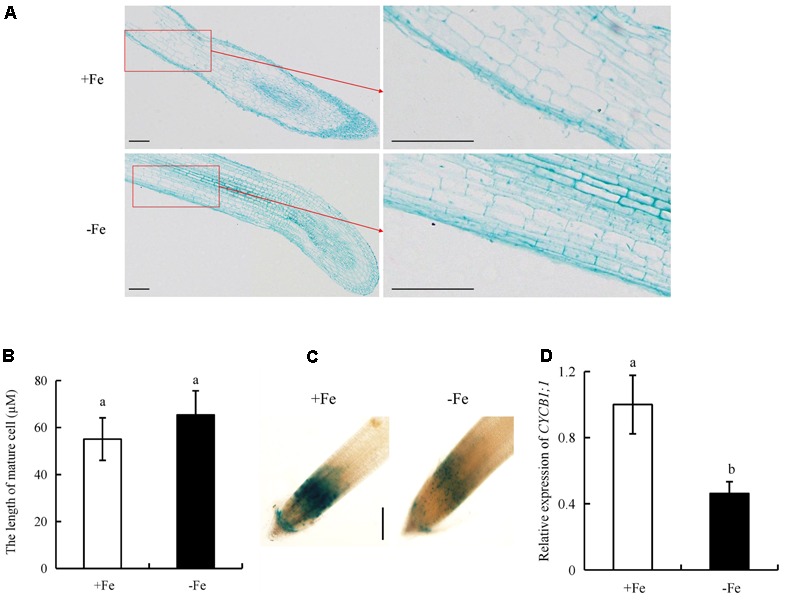
Epidermal cell lengths in the maturity zone, expression levels of *pCYCB1;1::GUS* and *OsCYCB1;1* in the meristem zone. Seedlings were grown in hydroponic media containing -Fe (0 μM) and +Fe (20 μM) for 14 days. **(A,B)** Epidermal cell length of seminal root. Bar = 100 μm. **(C)** Cell cycle activity of the root meristemof seminal root, as monitored by the *pCYCB1;1::GUS* reporter. Bar = 500 μm. **(D)** The expression of *CYCB1;1* gene. Data are means ± SE and bars with different letters indicate significant difference at *P* < 0.05 tested with ANOVA.

To determine the mechanism by which -Fe regulates root elongation, we analyzed the lengths of epidermal cells in the maturity zone (**Figures 9A,B**). The lengths of epidermal cells did not differ between -Fe and +Fe, suggesting that the inhibitory effect of Fe deficiency on root elongation was not due to changes in cell elongation.

We used transgenic plants expressing the *pCYCB1;1::GUS* construct to assess the cyclic activity of cells in the root meristem. *CYCB1;1::GUS* activity and *CYCB1;1* expression in the root meristem were decreased under -Fe relative to +Fe (**Figures 9C,D**). Therefore, -Fe inhibited root elongation primarily by regulating root meristem activity rather than the elongation of epidermal cells in the maturity zone.

## Discussion

The regulation of root growth and development in response to Fe deficiency is essential for the growth and productivity of plants. Fe deficiency exerts marked effects on root development in several plant species (Moog et al., 1995; Dasgan et al., 2002; Jin et al., 2008, 2009, 2011; Long et al., 2010; Zhai et al., 2016). In this study, LR density and seminal root length changed significantly as Fe concentration decreased from 20 to 0 μM (**Figure 1**). LR density increased by 30% and seminal root length decreased by 21% under Fe-deficient (-Fe) relative to control (+Fe), suggesting that Fe deficiency affects the root growth of rice.

Several environmental and endogenous signals may be integrated to regulate auxin distribution by influencing its synthesis and polar transport (Vanneste and Firml, 2009; Giehl et al., 2012). The elevated auxin level in roots under Fe-deficient is considered to be an adaptive strategy in *Arabidopsis* and tomato (Chen et al., 2010; Jin et al., 2011). In this study, auxin levels were significantly increased in the first leaves, but were decreased in shoot-root junctions and roots under Fe deficient (-Fe) (**Figure 2A**). [^3^H] IAA transport in roots was significantly decreased under -Fe, suggesting suppression of auxin transport from shoots to roots. These results differed from the findings in tomato (Chen et al., 2010) and *Arabidopsis* (Jin et al., 2011), possibly due to use of different plant species and growth conditions. For example, the treat time of this study (14 days) is longer than that of previous experiments (within 7 days). Moreover, the results of root after ttreatment with NPA and IAA suggested that seminal root elongation rather than LR formation is regulated by auxin level under Fe-deficient (-Fe). Auxin synthesis and transport in rice are regulated by *YUCCA* and *PIN* genes, respectively, in response to nutrient deficiency (Chen et al., 2013; Sun et al., 2014). In this study, the expression levels of *YUCCA1* and *YUCCA8* in the first leaves were upregulated, but that of *PIN1a-c* in roots was significantly downregulated under Fe- deficient (-Fe), suggesting that the -Fe-mediated increase in auxin biosynthesis in the first leaves is induced by *YUCCA1* and *YUCCA8* (**Figure 4A**), and the decreased polar auxin transport from shoot to root is regulated by *PIN1a-c* (**Figure 4B**).

Nitric oxide functions as a signaling molecule in regulating root formation in plants subjected to Fe deficiency (Jin et al., 2011; Meng et al., 2012). Jin et al. (2011) suggested that Fe deficiency inhibits elongation of primary roots in tomato. Fernández-Marcos et al. (2011) showed that NO decreases the length of primary roots in *Arabidopsis*. The promotion or inhibition of root elongation likely depends on the NO concentrations and experimental conditions used (Zhao et al., 2007; Manoli et al., 2014; Trevisan et al., 2014). NO inhibition of primary root elongation is in most cases detected following exogenous application of NO donors (Tomato, Correa-Aragunde et al., 2004; *Arabidopsis*, Méndez-Bravo et al., 2010; Fernández-Marcos et al., 2011; Bai et al., 2014) and in NO-overproducing mutants (*Arabidopsis*, He et al., 2004; Fernández-Marcos et al., 2011). In this study, 30 μM SNP increased the LR density, but decreased the seminal root length to levels similar to those under Fe-deficient (-Fe) (Supplementary Figure 8). The application of cPTIO under Fe-deficient (-Fe) resulted in a root morphology similar to that under control plants (+Fe), confirming that an appropriate amount of NO induces LR formation, but inhibits seminal root elongation of rice plants under Fe-deficient (-Fe).

NR and nitric oxide synthase-like (NOS-like) are involved in NO production in plants (Wilson et al., 2008). Moreau et al. (2008) demonstrated that *AtNOS1* did not affect NOS activity; it thus was renamed NO-associated enzyme (*AtNOA1*) in *Arabidopsis*. Compared with WT plants, the roots of a *noa1* mutant (formerly *Atnos1*) had lower NO levels (Guo and Crawford, 2005). In addition to the *NOA1*-dependent pathway, the findings of Bright et al. (2006) and Zhao et al. (2009) supported involvement of *NIA1* in NR-mediated NO production. In this study, NR activity, *NIA2* and *NOA* expression were significantly increased under Fe-deficient (-Fe) compared with control (+Fe). However, NO-associated green fluorescence, seminal root length, and LR density had no response to Tu treatment, but were significantly reduced by the NOS inhibitor L-NAME, and the change in the root morphology and NO-associated green fluorescence of *nia2* mutants in response to Fe deficiency was similar to that of the WT (Supplementary Figures 7, 8), suggesting that the NOS-like pathway rather than NR pathway is involved in regulation of root growth under Fe deficiency.

Several lines of studies suggested that NO and auxin in regulating Fe-acquisition by two strategies (Chen et al., 2010; García et al., 2011; Jin et al., 2011; Blum et al., 2014). Firstly, auxin and NO increased the Fe uptake by up-regulating the related genes in plants (Chen et al., 2010; García et al., 2011; Blum et al., 2014). Chen et al. (2010) suggested that NO acts downstream of auxin to trigger root Ferric-Chelate Reductase activity by increasing the expression of *FIT* and *FRO2* genes under Fe deficiency. García et al. (2011) demonstrated that NO and ethylene influence the production of the other and that both substances could be necessary for up-regulating Fe-acquisition genes. Blum et al. (2014) showed that the expression of *IRT1* in roots was modulated by ethylene–auxin interplay. These results indicated that auxin, NO and ethylene form a regulatory triangle to enhance Fe uptake. Secondly, auxin and NO increased the Fe-acquisition by modulating root formation (Nacry et al., 2005; Wang et al., 2010; Jin et al., 2011). NO acted downstream of auxin in regulation of Fe acquisition by increasing branching root formation under Fe-deficient (Jin et al., 2011). In this study, NO inhibited auxin polar transport from shoot to roots under Fe deficiency (**Figure 8A**). However, application of IAA did not affect the NO levels in root (Supplementary Figure 9). These results suggested that the interactions between signal molecule and phytohormone in response to Fe-deficient are more complex. Under control (+Fe), SNP completely restored the NPA-induced decrease in LR density, but IAA did not reverse the inhibitory effect of cPTIO under Fe-deficient (-Fe). These results verify NO acted downstream of auxin in regulating LR formation under Fe deficient plants (-Fe). This is consistent with the work of Jin et al. (2011). Fernández-Marcos et al. (2011) showed that NO suppressed primary root elongation by decreasing the auxin level in RTs, suggesting that the interaction effect of NO and auxin on root growth differs between elongation of roots and LR formation. In this study, IAA co-incubation partly restored the SNP-mediated decrease in seminal root elongation under control plants (+Fe), and NPA completely restored the positive effects of cPTIO on seminal root elongation under Fe-deficient (-Fe) (**Figure 8**), and the results of seminal root length in *ospin1b* mutant in response to IAA, SNP, and cPTIO treatments confirmed that the root elongation is dependent on the auxin levels in RTs of rice plants under Fe deficiency.

Root elongation is dependent on two basal development processes: cell division in the RT meristem and elongation of root cells in the maturity zone (Scheres et al., 2002). The activity of meristematic cells in root meristem affects root elongation (Blilou et al., 2005). Fernández-Marcos et al. (2011) reported that higher NO levels reduce root meristem activity in *Arabidopsis*. However, WT plants with NO depletion and a NO-deficient mutant exhibited reduced primary root elongation and had small root meristems (Sanz et al., 2014). In this study, Fe deficient plants (-Fe) decreased *pCYCB1;1::GUS* construct and *CYCB1;1* expression levels in RTs, but did not affect the length of mature cells (**Figure 9**). These findings suggest that seminal root elongation was regulated by decreasing cell division in the root meristem zone under Fe-deficient (-Fe).

## Conclusion

Nitric oxide was generated mainly by the NOS-like pathway and acted downstream of auxin in regulating LR formation, but NO inhibited root elongation by decreasing the auxin level in RTs under Fe-deficient (-Fe). Fe deficiency affected root elongation mainly by decreasing root meristem activity rather than elongation of epidermal cells in the maturity zone (Supplementary Figure 11).

## Author Contributions

HS performed experiments. FF assisted the experiment. JL analyzed data. QZ designed the experiment and wrote the paper.

## Conflict of Interest Statement

The authors declare that the research was conducted in the absence of any commercial or financial relationships that could be construed as a potential conflict of interest.
