# Supplementary material for: The Interaction between Auxin and Nitric Oxide Regulates Root Growth in Response to Iron Deficiency in Rice
Source: Front Plant Sci. 2017 Dec 22;8:2169. doi: 10.3389/fpls.2017.02169 (PMC5743679; doi:10.3389/fpls.2017.02169)
Supplement: Supplementary file 1 [file Image_1.PDF]

Supplementary Table 1 The primers for qRT-PCR of *PIN* family genes

Gene	Primer sequence
OsPIN1a	5'-TCATCTGGTCGCTCGTCTGC-3'
	5'-CGAACGTCGCCACCTTGTTC-3'
OsPIN1b	5'-TGCACCCTAGCATTCTCAGCA-3'
	5'-CCCTCCTCCCAAATTCTACTT-3'
OsPIN1c	5'-CCGTCAGGTTCCTCGTGGGT-3'
	5'-TCACGGCTGTGCTCAGAATG- 3'
OsPIN2	5'-CAACACCTACTCCAGCCTC-3'
	5'-TGGACCAGTCAAGAACCTC-3'
OsPIN5a	5'-GGGGCTGGTGCTAAAGTTCG-3'
	5'-TGAGGTAGGGCTGCCTGTATG-3'
OsPIN5b	5'-GGGCAGCAGGAGAGGGTGATAG-3'
	5'-GAATCGGCAGAGAGATCAATGT-3'
OsPIN9	5'-GATACAAGATAGCGTCGTTCTC-3'
	5'-ATGATGTCTGCGTGGACCT-3'
OsPIN10a	5'-GTTGGATTGAGATAGGCTGAGGAG-3'
	5'-ATGGCGACGAAGCGGTTGAT-3'
OsPIN10b	5'-TCCGATGCAGGGTTAGGC-3'
	5'-AGGATGGTAGCGTGGAGGTT-3'
OsACTIN	5'-CAACACCCCTGCTATGTACG-3'
	5'-CATCACCAGAGTCCAACACAA-3'

Supplementary Table 2 The primers for qRT-PCR of *YUCCA* family genes

Gene	Primer sequence
OsYUCCA1	5'-AGGTGTTGGTCGTGGGATGCG-3'
	5'-GCGATGCCGAACGTGGATAGA-3'
OsYUCCA2	5'-TATGGATCGGCAACCATTTGA-3'
	5'-CGCTGGGAAGACTGTCCTTGT-3'
OsYUCCA3	5'-GGAAGCGTGTTCTCGTTGTTG-3'
	5'-ACATTGACAGCCCAAAGGTGG- 3'
OsYUCCA4	5'-CCTCGACCTCTGCAACCACAATG-3'
	5'-CGACAACAGGAGTACCAGCCAATC-3'
OsYUCCA5	5'-GTCAGCCTCGACCTCTGCAACA-3'
	5'-TGGGAAACCACTTGAGAAGGAACAC-3'
OsYUCCA6	5'-GGATACCAAAGCAACGTCCCC-3'
	5'-TGAAGCCAACAGAGTAGAGCCCTG-3'
OsYUCCA7	5'-ACCGGCTACCGCAGCAATGTG-3'
	5'-CGTACAGCCCCGACTCACCCT-3'
OsYUCCA8	5'-GAGATGTGCCTGGACCTCTGC-3'
	5'-GTGTCTCCCAGCACCATCCTT-3'

Supplementary Table 3 The primers for qRT-PCR of *NOA*, *NIA1*, *NIA2*, *CYCB1;1* genes

Gene	Primer sequence
OsNOA	5'-TGCTTCTGTGGTTGGGAC-3'
	5'-TCTAAGGGCACGGTGTTT-3'
OsNIA1	5'-CCAATTCTTTCATCGTGTTCT-3'
	5'-CATGCAGCATTTCGTTTCT-3'
OsNIA2	5'-ACTGGTGCTGGTGCTTCTGG-3'
	5'-CGGCTGGGTGTTGAGGGACT-3'
OsCYCB1;1	5'-CACGTCGACTATAGACTAAGCCATTGAGGCGTAT -3'
	5'-AAAGGTACCAGAGCTGATCTCGATGACATGCTCGG-3'

Supplementary Table 4 The primers for qRT-PCR of *YSL15* and *IRT1* family genes.

Gene	Primer sequence
OsYSL15	5'-GAGCTTCGCCATCGACTAT-3'
	5'-TTGTTCATCTTGTTCCAAGCA-3'
OsIRT1	5'-AGGTCGGTGCTCGTCTCT-3'
	5'-TGTCCCTGTACACCCTGGTC-3'

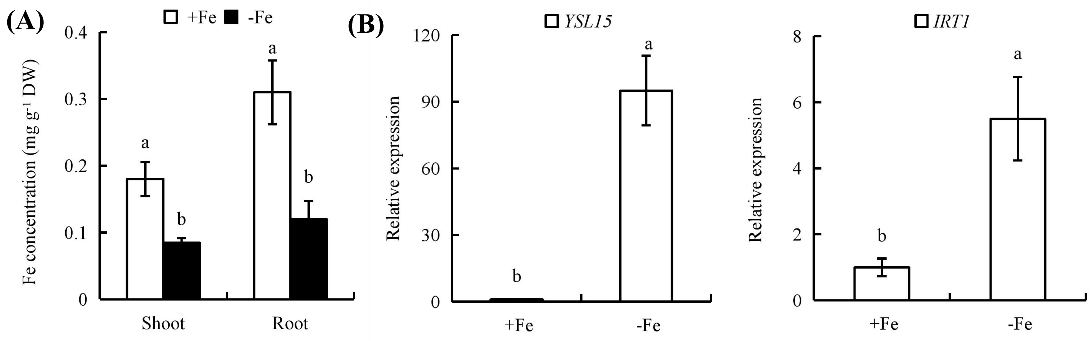

Supplementary FIGURE 1 Fe concentration and qRT-PCR analysis of *YSL15* and *IRT1* genes in the rice seedlings. Seedlings were grown in hydroponic medium containing -Fe (0 μM) and +Fe (20 μM) for 14 days. (A), Fe concentrations of shoot and root. (B), Relative expression of *YSL15* and *IRT1* in roots. Data are means ± SE and bars with different letters indicate significant difference at $P < 0.05$ tested with ANOVA.

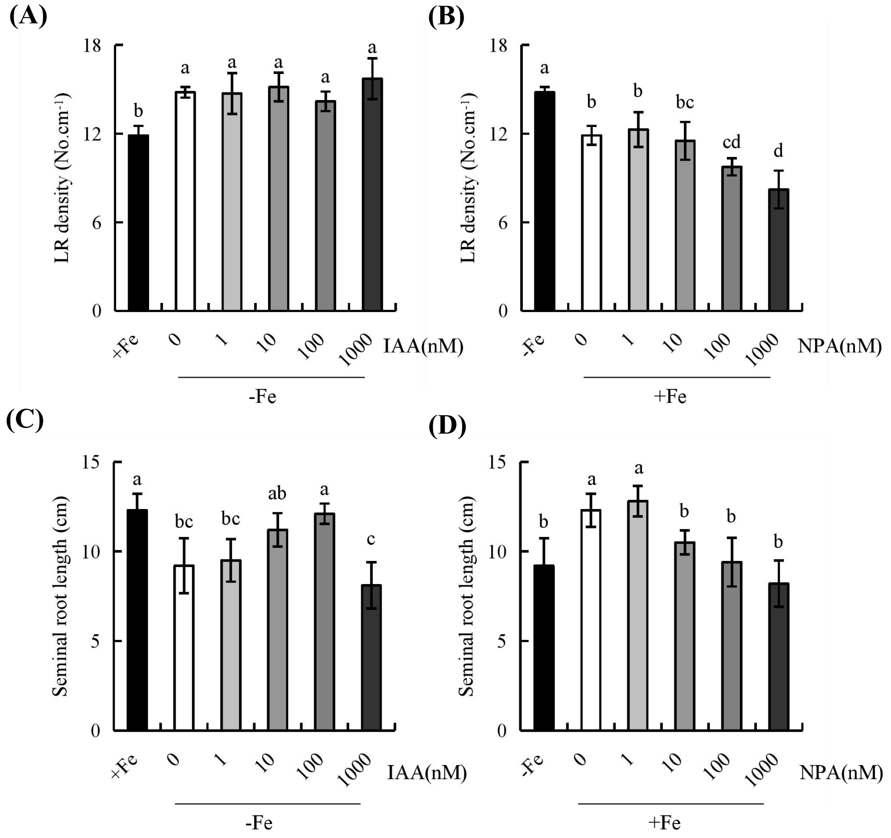

Supplementary FIGURE 2 The effects of IAA and NPA on root architecture of rice seedlings. Seedlings were grown in hydroponic medium containing -Fe (0 μM) and +Fe (20 μM) in addition to varying IAA (0-1000nM) and NPA (0-1000nM) concentrations for 14 days. (A-B), LR density. (C-D), Seminal root length. Data are means ± SE and bars with different letters indicate significant difference at $P < 0.05$ tested with ANOVA.

(A) (B)

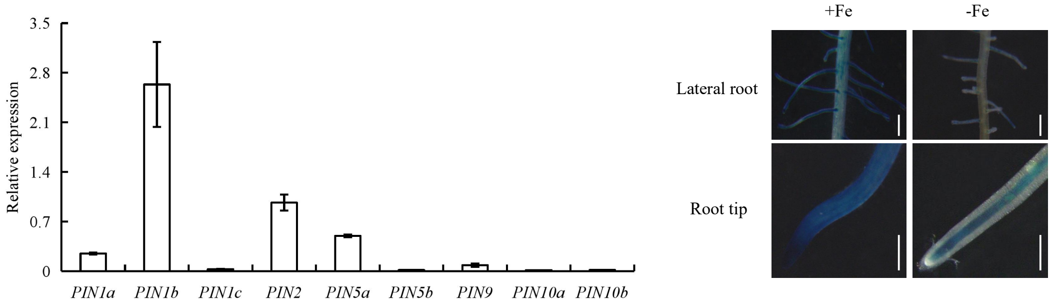

Supplementary FIGURE 3 qRT-PCR analysis of *PIN* family genes of roots in the wild-type (WT) rice seedlings and histochemical localization of *proPIN1b::GUS* activity in roots. Seedlings were grown in hydroponic media containing -Fe (0 μM) and +Fe (20 μM) for 14 days. (A), Relative expression of *PIN* family genes. (B), Histochemical localization of *proPIN1b::GUS* activity. Plants were stained for *GUS* activity in roots for 2 h at 37°C. Bar=1mm. Data are means ± SE and bars with different letters indicate significant difference at P < 0.05 tested with ANOVA.

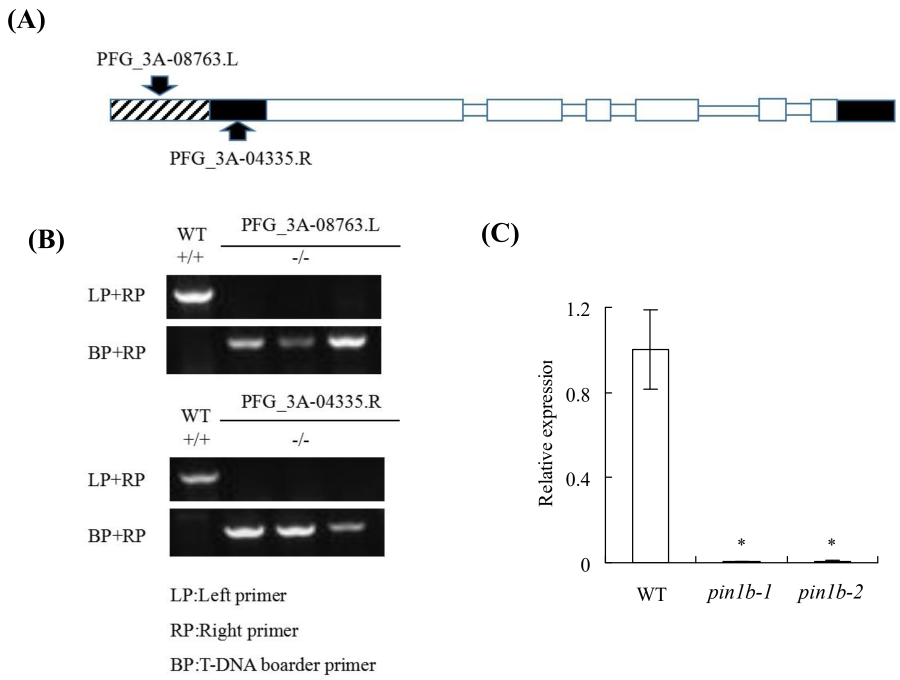

Supplementary FIGURE 4 Identification of T-DNA insertion *ospin1b* mutants (*ospin1b-1* and *ospin1b-2*). (A), Gene structure of *OsPIN1b* and the T-DNA insertion sites. The exons and introns are indicated by white boxes and lines, respectively; the promoter and URT regions are indicated by striated box and bold boxes, respectively. (B) Identification of homozygous T-DNA insertion mutants by two rounds of PCR. (C) qRT-PCR analysis for the expression of *OsPIN1b* in roots of WT and *ospin1b-1* (PEG_2A-08763.L) and *ospin1b-2* (PEG_3A-04335.R) mutants. Data are means ± SE from three replicates. *, $P < 0.05$ (ANOVA) comparing WT plants and two *ospin1b* mutants.

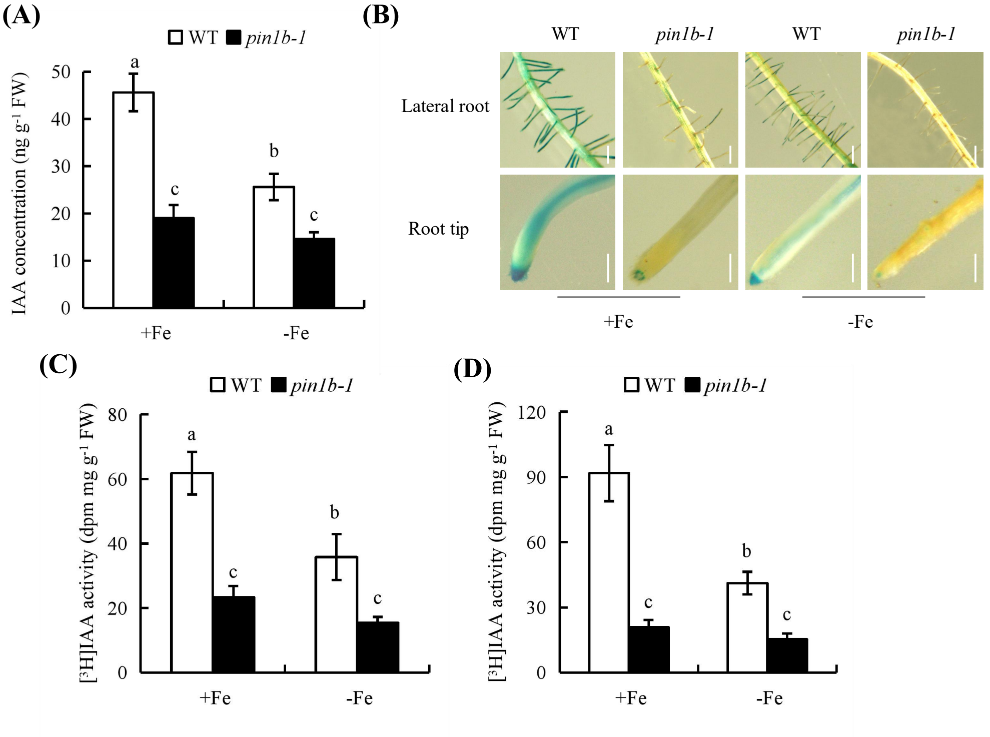

Supplementary FIGURE 5 IAA concentration and histochemical localization of *DR5::GUS* activity and [^{3}H]IAA transport in wild-type (WT, Donjin) and *ospin1b-1* mutant. Rice seedlings were grown in hydroponic media containing -Fe (0 μM) and +Fe (20 μM) for 14 days. (A) IAA concentration in roots. (B), *DR5::GUS*, a specific reporter that contains seven repeats of a highly active synthetic auxin response element and can reflect the *in vivo* auxin level. Plants were stained for *GUS* activity in the first leaf for 24 h and in other regions for 2h at 37°C. Bar=1mm. [^{3}H]IAA transport in lateral root zone (C) and root tip (D). Data are means ± SE and bars with different letters indicate significant difference at $P < 0.05$ tested with ANOVA.

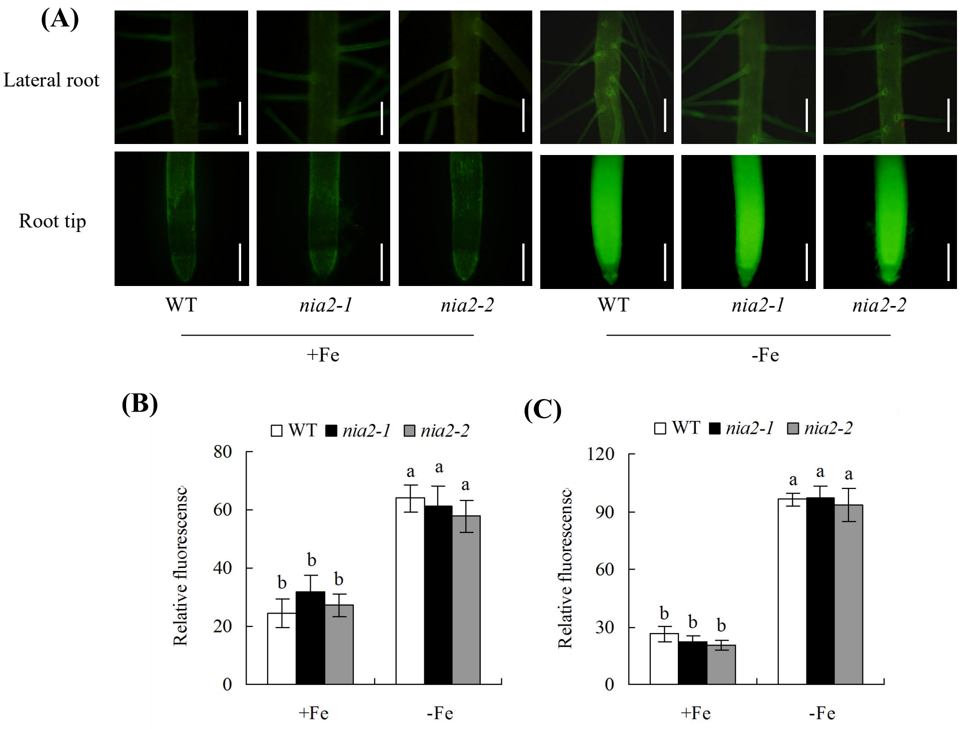

Supplementary FIGURE 6 Accumulation of nitric oxide (NO) and root morphology in wild-type (WT, Donjin) and *nia2* mutants. Seedlings were grown in hydroponic medium containing -Fe (0 μM) and +Fe (20 μM) for 14 days. (A), Photographs of NO production shown as green fluorescence in the roots, and NO production expressed as fluorescence intensity relative to lateral root zone (B) and root tip (C). Bar=1mm. Data are means ± SE and bars with different letters indicate significant difference at $P < 0.05$ tested with ANOVA.

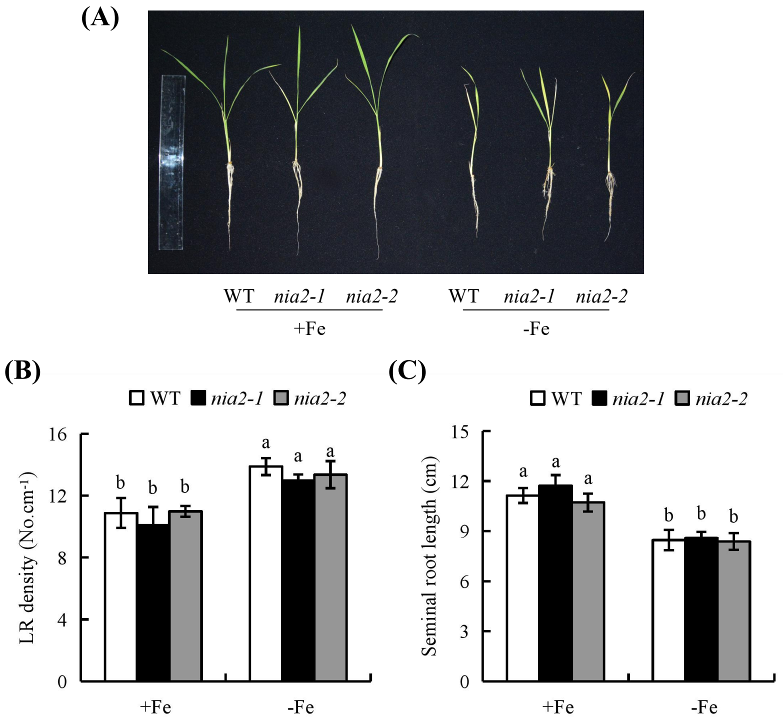

Supplementary FIGURE 7 The root morphology in wild-type (WT, Donjin) and *osnia2* mutants. Seedlings were grown in hydroponic media containing -Fe (0 μM) and +Fe (20 μM) for 14 days. (A), The morphology of the rice plants. (B), LR density. (C), The length of seminal root. Data are means ± SE and bars with different letters indicate significant difference at $P < 0.05$ tested with ANOVA.

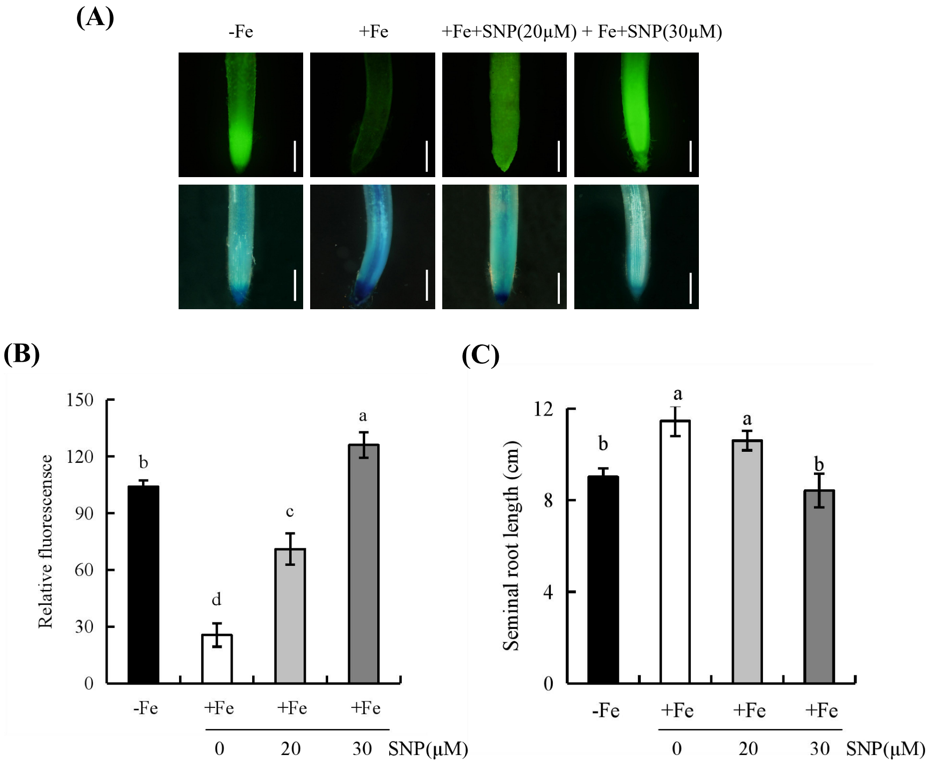

Supplementary FIGURE 8 The accumulation of nitric oxide (NO), histochemical localization of *DR5::GUS* activity, and seminal root length of rice seedlings. Seedlings were grown in hydroponic medium containing -Fe (0 μM) and +Fe (20 μM) in addition to SNP (0-30 μM) for 14 days. (A-C), Photographs of NO production shown as green fluorescence and histochemical localization of *DR5::GUS* activity in the root tip (A), and NO production expressed as fluorescence intensity in root tip (B). (C), Seminal root length. Bar=1mm. Data are means ± SE and bars with different letters indicate significant difference at $P < 0.05$ tested with ANOVA.

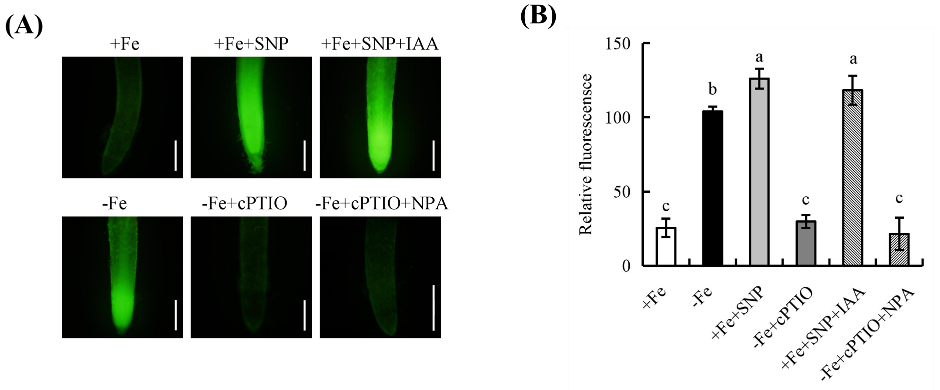

Supplementary FIGURE 9 The accumulation of nitric oxide in the rice seedlings. Seedlings were grown in hydroponic medium containing -Fe (0 µM) and +Fe (20 µM) in addition to IAA (100nM), NPA (100nM), SNP (30 µM) and cPTIO (80 µM) for 14 days. Photographs of NO production shown as green fluorescence (A), and NO production expressed as fluorescence intensity in root tip (B). Bar=1mm. Data are means ± SE and bars with different letters indicate significant difference at $P < 0.05$ tested with ANOVA.

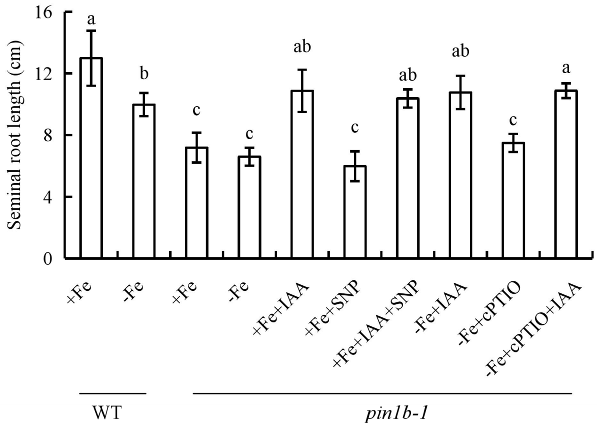

Supplementary FIGURE 10 Seminal root length of wild-type (WT, Donjin) and *ospin1b-1* mutant. Seedlings were grown in hydroponic medium containing -Fe (0 μM) and +Fe (20 μM) in addition to IAA (100nM), NPA (100nM), SNP (30 μM) and cPTIO (80 μM) for 14 days. Data are means ± SE and bars with different letters indicate significant difference at $P < 0.05$ tested with ANOVA.

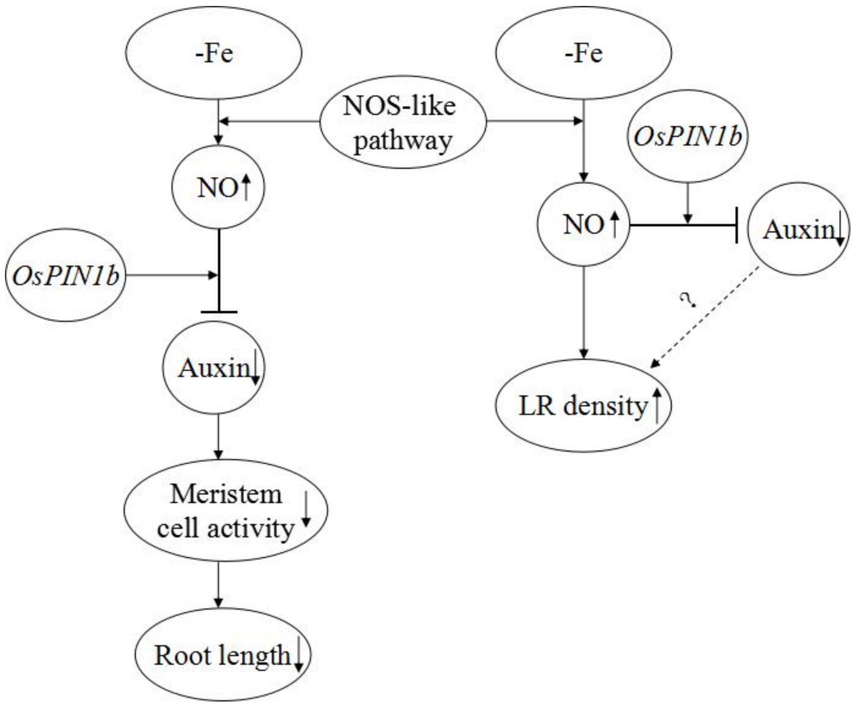

Supplementary FIGURE 11 Schematic model of the interaction between auxin and nitric oxide (NO) regulates root growth under Fe deficiency in rice. Solid arrows denote regulatory pathways and the dashed arrows present a possible pathway.NOS, Nitric Oxide Synthase; -Fe (0 μM) ; LR, Lateral root.
